# Development of Multifunctional Catalysts for the Direct Hydrogenation of Carbon Dioxide to Higher Alcohols

**DOI:** 10.3390/molecules29112666

**Published:** 2024-06-04

**Authors:** Yun Chen, Jinzhao Liu, Xinyu Chen, Siyao Gu, Yibin Wei, Lei Wang, Hui Wan, Guofeng Guan

**Affiliations:** 1 State Key Laboratory of Materials-Oriented Chemical Engineering, College of Chemical Engineering, Jiangsu National Synergetic Innovation Center for Advanced Materials, Nanjing Tech University, Nanjing 210009, China; chenyunnjtech@163.com (Y.C.); ljzsatori@outlook.com (J.L.); cxy702@njtech.edu.cn (X.C.); cxlcwgsy@163.com (S.G.); guangf@njtech.edu.cn (G.G.); 2 State Key Laboratory of High-Efficiency Utilization of Coal and Green Chemical Engineering, School of Chemistry and Chemical Engineering, Ningxia University, Yinchuan 750021, China; yibin.wei@nxu.edu.cn

**Keywords:** CO_2_ hydrogenation, higher alcohol, multifunctional catalysts, C-C coupling, reaction mechanism

## Abstract

The direct hydrogenation of greenhouse gas CO_2_ to higher alcohols (C_2+_OH) provides a new route for the production of high-value chemicals. Due to the difficulty of C-C coupling, the formation of higher alcohols is more difficult compared to that of other compounds. In this review, we summarize recent advances in the development of multifunctional catalysts, including noble metal catalysts, Co-based catalysts, Cu-based catalysts, Fe-based catalysts, and tandem catalysts for the direct hydrogenation of CO_2_ to higher alcohols. Possible reaction mechanisms are discussed based on the structure–activity relationship of the catalysts. The reaction-coupling strategy holds great potential to regulate the reaction network. The effects of the reaction conditions on CO_2_ hydrogenation are also analyzed. Finally, we discuss the challenges and potential opportunities for the further development of direct CO_2_ hydrogenation to higher alcohols.

## 1. Introduction

Since the Industrial Revolution, with the increasing human demand for energy, fossil fuels such as coal, oil, and natural gas have been intensively exploited [1]. However, the excessive emissions of greenhouse gases such as CO_2_ leads to environmental problems such as the greenhouse effect and ocean acidification [2,3]. To cope with this problem, it is necessary not only to reduce emissions but also develop our knowledge of carbon capture, utilization, and storage (CCUS) [4,5]. Converting CO_2_ into various high-value chemicals such as alkanes, olefins, alcohols, and aromatics can alleviate our reliance on fossil fuels, which is of great significance for the sustainable development of the energy and chemical industries [6,7,8,9,10]. Due to the chemical inertness of the CO_2_ molecule, the conversion of CO_2_ to methanol and C_2+_ products usually requires a large energy input under harsh reaction conditions, such as high temperature and pressure [11]. Introducing another substance with higher Gibbs free energy as a co-reactant, such as green H_2_, will make this reaction thermodynamically easier [12]. Therefore, the hydrogenation of CO_2_ to various value-added products is one of the promising methods for utilizing this carbon-rich resource [6,13,14,15,16,17,18,19,20,21]. The main technologies for producing high-value chemicals from CO_2_ include thermal catalytic methods, photocatalytic methods [22,23,24,25], and electrocatalytic methods [26,27,28,29]. Among them, thermal catalytic CO_2_ conversion technology has the advantages of a high catalytic performance, easy-to-control reaction conditions, and large-scale industrial application. It has, therefore, received widespread attention in the past few decades.

The term “higher alcohols” generally refers to C_2+_ alcohols [30]. Due to their high energy density and low vapor pressure, higher alcohols can be used directly as fuel or fuel additives [31]. Higher alcohols are also an important raw or intermediate material for the production of other chemical products [32]. They have widespread applications in different fields, such as energy, biology, and chemical engineering [33]. At present, higher alcohols are mainly produced through the fermentation of crops such as corn and sugarcane or the hydration of alkenes [34]. However, the former consumes a large amount of grain, while the latter relies on petroleum resources and has a low single-pass conversion [35]. Therefore, the utilization of industrial emissions of CO_2_ and renewable hydrogen energy to produce higher alcohols is of great significance.

The direct conversion of CO_2_ hydrogenation to higher alcohols is challenging due to the existence of various parallel and sequential reactions, which makes the reaction network complex. The products of CO_2_ hydrogenation are also complicated [36,37,38], such as C1 (e.g., CH_3_OH, CO, and CH_4_) and C_2+_ products (e.g., alkanes, alkenes, and higher alcohols) [39,40]. Table 1 provides an overview of the principal reactions involved in the process of CO_2_ hydrogenation, along with their corresponding reaction enthalpies. With the exception of the reverse water gas shift (RWGS) reaction, most reactions exhibit an exothermic behavior, indicating thermodynamic favorability under low temperatures [41]. The RWGS reaction is unlikely to occur independently, and it may proceed as a parallel side-reaction. At a low temperature, the formation of CO through the RWGS reaction can be restrained, but the inertness of CO_2_ makes it difficult to activate. Thus, the reaction conditions must be carefully explored to obtain the target product with a high yield.

The equilibrium constants of alkanes are much higher than those of alcohols under all temperatures; thus, alkanes are thermodynamically favored. Methane and other hydrocarbon products occur as main products in many catalytic systems. The ethanol synthesis reaction exhibits a lower Gibbs free energy and a larger equilibrium constant compared to methanol synthesis, rendering it more favorable thermodynamically. However, due to the shared active sites involved, the synthesis of alcohol with different carbon numbers remains kinetically competitive where methanol is usually the primary product [42].

The study of CO_2_ hydrogenation to higher alcohols can be traced back to the 1980s [43], but most catalysts are directly modified from the conversion of syngas to alcohols by hydrogenation. The problems of low activity and limited selectivity hinder its practical application in industry [44]. Due to the co-existence of various intermediates, the reaction mechanism for CO_2_ hydrogenation to higher alcohols is still controversial. However, it is generally accepted that higher alcohols are formed through multiple sequential steps, such as CO_2_ activation, carbon chain growth, and alcohol generation. Thus, a single active site is unlikely to catalyze the entire catalytic cycle. Therefore, catalysts with multifunctional sites hold the potential to combine the above sequential steps for higher alcohol synthesis. Thus, the rational design of multifunctional catalysts with a controlled reaction channel is crucial for the selective synthesis of target products. In addition, the reaction-coupling strategy holds good potential to yield more value-added products. For example, the combination of methanol synthesis with Fischer–Tropsch synthesis (FTS) by thermocatalysis produces more higher alcohols. Recently, cascade electrochemical CO_2_ reduction with thermocatalysis or biocatalysis has also been used to produce high-value chemicals, such as propionaldehyde, butane, and glucose [45,46,47,48].

The hydrogenation of CO/CO_2_ into high-value C_2+_ chemicals has drawn the attention of many researchers, as it is important for achieving carbon neutrality. However, the complicated reaction networks, coupled with multiple intermediates and reaction pathways, make selective control difficult; therefore, conventional catalysts still suffer from limited product selectivity. Recently, with the development of catalyst preparation and characterization technologies, remarkable progress has been made in the area of higher-alcohol synthesis (HAS) from CO_2_ hydrogenation. This manuscript provides a comprehensive summary of recent advances in the development of highly efficient catalysts for CO_2_ hydrogenation to higher alcohols. Compared with continuous fixed-bed reactors, the HAS selectivity of catalysts evaluated in a batch tank reactor is often higher due to the solvent effect. However, a batch tank reactor cannot be operated continuously, and most recent catalysts are evaluated in fixed-bed reactors. In this work, the catalytic performances of the multifunctional catalysts are evaluated in fixed-bed reactors, if not specifically indicated. The catalysts are usually loaded into the middle of a fixed-bed reactor with flowing feeding gas (CO_2_ and H_2_), and the products are analyzed online or offline by a gas chromatograph (GC) equipped with thermal conduction detectors and flame ionization detectors.

This work is divided into the following main sections: (1) The latest research progress on noble metal catalysts, Co-based catalysts, Cu-based catalysts, Fe-based catalysts, and tandem catalysts for CO_2_ hydrogenation to higher alcohols is summarized. (2) Possible reaction mechanisms for CO_2_ hydrogenation to higher alcohols on multifunctional catalysts are discussed. (3) The effects of reaction conditions such as temperature, pressure, space velocity, H_2_/CO_2_ ratio, and relative humidity on the performance of the catalysts are analyzed. The main contributions of this study are as follows: (1) a comprehensive review of research progress on the hydrogenation of CO_2_ to higher alcohols using multifunctional catalysts; (2) a summary of possible reaction mechanisms for the hydrogenation of CO_2_ to higher alcohols; (3) a detailed discussion of the effects of reaction conditions on catalytic performance and catalyst stability; and (4) an outline of the challenges and opportunities in the hydrogenation of CO_2_ to higher alcohols, providing important guidelines for the further development of HAS.

## 2. Multifunctional Catalysts for CO_2_ Hydrogenation to Higher Alcohols

As mentioned above, an effective higher-alcohol synthesis (HAS) catalyst should promote CO_2_ activation, C-C coupling, and other elementary reactions. To address this challenge, significant efforts have been made in the development of catalysts, including noble metal catalysts, Co-based catalysts, Cu-based catalysts, and Fe-based catalysts [49,50,51]. Recently, the reaction-coupling catalytic strategy has drawn the attention of many researchers. Multifunctional catalysts combine the advantages of catalysts with different functions. The formation or migration of key intermediates (CO*/CH_x_O*) can be manipulated, thereby significantly improving the production of higher alcohols. Therefore, after the introduction of noble metal catalysts, Co-based catalysts, Cu-based catalysts, and Fe-based catalysts, this paper focuses on the state of the art of tandem catalysts.

### 2.1. Noble Metal Catalysts

Supported noble metal catalysts (Rh, Pd, Pt, etc.) have received extensive attention in the field of CO_2_ hydrogenation reactions due to their good C-C coupling ability, which is conducive to CO_2_ hydrogenation (Table 2). The types and forms of noble metal-based catalysts have a greater impact on CO_2_ hydrogenation reactions. Rh-based catalysts have been extensively studied in relation to ethanol synthesis from syngas, prompting researchers to explore the use of Rh catalysts for CO_2_ hydrogenation to produce higher alcohols [52,53,54,55].

Researchers have found that Rh-TiO_2_, RhNa-TiO_2_, and Rh-SiO_2_ catalysts have a good performance in CO_2_ hydrogenation [56,57,58]. Inoue et al. [56] prepared different supported Rh-based catalysts and discussed the effect of the support on hydrogenation of CO_2_. The product was almost exclusively methane over Rh/MgO and Rh/ZrO_2_ catalysts. In the case of the Rh/TiO_2_ catalyst, its selectivity to methanol was the highest of all the catalysts. Thus, selectivity to methanol is considered to be enhanced by easily reducible supports such as TiO_2._ Bando et al. [57] performed CO_2_ hydrogenation over Li-promoted Rh ion-exchanged zeolite catalysts (Li/RhY). They found that the selectivity for methane was almost 100% for the undoped catalyst (RhY). After modification with the alkali metal Li, the formation of methane was suppressed, while the selectivity of CO increased. Moreover, the promoted formation of methanol and ethanol was also observed. The addition of the alkali metal could increase the amount of CO intermediates, which then would react with *CH_3_ to form ethanol. In order to further enhance the C-C coupling ability of Rh-based catalysts, transition metals were also introduced into Rh-based catalysts to promote the formation of C_2+_ products [59,60].

Liu et al. [61] embedded Rh onto a Ti-doped CeO_2_ support to construct a Rh_1_/CeTiO_x_ single-atom catalyst, which exhibited high ethanol selectivity. The oxygen vacancy-Rh Lewis acid–base pairs are conducive to CO adsorption and activation. The C-O bond in CH_x_OH* and COOH is cleaved into CH_x_* and CO* substances, and then C-C coupling and hydrogenation generate ethanol. The strong Rh-O bond generated by Ti-doping-induced crystal reconstruction contributes to the stability of the catalyst.

Aside from typical Rh-based catalysts, Pd, Pt, and Au have also been investigated in terms of CO_2_ hydrogenation to HAS. Liu et al. [62] synthesized a dual-atom Pd/CeO_2_ catalyst with high ethanol selectivity (97.8%). Unfortunately, the diatomic Pd active sites in the catalyst were easily aggregated into Pd particles. The activity of the catalyst declined quickly, and the stability of the catalyst was poor. The product selectivity also shifted from ethanol to methanol and CO. To solve this problem, a nano-reactor (Pd_2_Ce@Si_16_) with a hydrophobic shell was prepared. Diatomic Pd active sites can be stabilized through the microenvironment created by the in situ enrichment of water. In addition, in order to study the influence of the morphology of the support on the catalyst, Bi et al. [63] used Co_3_O_4_ with different morphologies (nanorods and nanoplates) to support Pt nanoparticles with prepared Pt/Co_3_O_4_ catalysts. The active surface of the Pt/Co_3_O_4_-p catalyst consisted of highly dispersed Pt and Co nanoparticles anchored on a Co_3_O_4_ support with some oxygen vacancies. The synergic effect of the Pt and Co nanoparticles and the oxygen vacancies of Co_3_O_4_-x improved the adsorption of H_2_ and CO_2_ and achieved the highest C_2+_OH yield of 0.56 mmol g_cat_^−1^ h^−1^ at 200 °C and 2 MPa. Some researchers have found that the interaction between multiple metal components can regulate the activity and selectivity of the catalyst. He et al. [64] used the co-precipitation method to synthesize heterogeneous PtRu/Fe_2_O_3_, where Pt and Ru bimetallic catalysts were supported on Fe_2_O_3_. The hybrid sites created from the synergistic combination of Pt and Ru resulted in higher activity and selectivity.

However, the high cost of Rh and other precious metal catalysts limits their industrialization. Due to strong CO adsorption, the CO poisoning of precious metals in HAS should be investigated, although no researchers have paid attention to this problem [65,66].

**Table 2:** Catalytic performance of HAS from CO_2_ hydrogenation over noble metal catalysts.

Enter	Catalysts	T (°C)	P (MPa)	GHSV ^a^ /L g^−1^ h^−1^	H_2_ /CO_2_	X ^b^_CO2_ (%)	S ^c^_CO_ (%)	S ^d^_HC_ (%)	S ^e^_MeOH_ (%)	S ^f^_HA_ (%)	STY ^g^_HA_ (mmol g_cat_^−1^ h^−1^)	Refs.
1	Rh-TiO_2_	300	10	6	3	/	/	69.8	21.1	9.3	0.7	[52]
2	RhLi-SiO_2_	240	5	6	3	7.0	15.7	63.5	5.2	15.5	0.36	[57]
3	Rh_1_/CeTiO_x_^h^	250	3	/	2	6.3	/	/	/	99.1	5.73 ^j^	[61]
4	1Pd_2_Ce@Si_16_	250	3	3	/	5.9	/	/	/	98.7	11.6 ^j^	[62]
5	Pt/Co_3_O_4_-p	200	2	6	3	22.4	/	80.1	19.9 ^i^		0.56	[63]
6	PtRu/Fe_2_O_3_^h^	200	20	/	/	/	0.4	57.3	6.3	36.0	2.4	[64]

^a^ Gas hourly space velocity; ^b^ CO_2_ conversion; ^c^ selectivity to carbon monoxide; ^d^ selectivity to hydrocarbon; ^e^ selectivity to methanol; ^f^ selectivity to higher alcohols; ^g^ space–time yield of higher alcohols; ^h^ tank reactor; ^i^ selectivity to total alcohols; and ^j^ space–time yield of ethanol.

### 2.2. Co-Based Catalysts

As a classical FTS catalyst, Co is commonly employed in CO hydrogenation to higher alcohols [67,68,69]. However, Co-based catalysts possess a strong hydrogenation capability [70,71,72], which leads to methane as the dominant product and exhibits a low activity for the RWGS. Adjusting the oxidation state of Co to form the Co-Co^δ+^ active phase is important to improve alcohol selectivity for Co-based catalysts. This is because Co^0^ tends to cause C-O bond dissociation to form *CH_x_ due to its strong hydrogenation ability, while Co^δ+^ can inhibit methanation and promote the formation of higher alcohols. It is important to avoid the complete reduction of Co^δ+^ to Cu^0^ under the reducing atmosphere, and how to maintain the stability of Co^δ+^ species (e.g., Co_3_O_4_, Co_2_C, etc.) is also crucial.

Arakawa et al. [73] first reported the use of a Co-based catalyst in CO_2_ hydrogenation to ethanol, with an ethanol selectivity of 7.9%. Afterwards, researchers began to use Co-based catalysts for HAS (Table 3). The accurate regulation of the electronic state and active species of Co-based catalysts seem to be challenging for HAS. The presence of alkali improves the carbon chain’s growth ability, promoting RWGS activity and suppressing methanation. Davis et al. [74] prepared Na-promoted cobalt oxide catalysts using SiO_2_ as a support (1%Na-20%Co/SiO_2_) and studied the effects of different pretreatment conditions on the active species of Co. After reduction with H_2_ at 350 °C, Co_3_O_4_ was completely reduced to metallic Co, and methane was the main product. When the reduction temperature was lowered to 250 °C, Co_3_O_4_ was reduced to CoO, and the hydrogenation ability of the catalyst was weakened, resulting in decreased CH_4_ selectivity. After CO reduction, the CoO and Co_2_C phases were generated, with CH_4_ selectivity further reduced to 15.3% and alcohol selectivity increased to 73.2%, indicating that Co_2_C may be a key species for inhibiting methane reaction and improving total alcohol selectivity.

**Table 3:** Catalytic performance of HAS from CO_2_ hydrogenation over Co-based catalysts.

Enter	Catalysts	T (°C)	P (MPa)	GHSV ^a^ /L g^−1^ h^−1^	H_2_ /CO_2_	X ^b^_CO2_ (%)	S ^c^_CO_ (%)	S ^d^_HC_ (%)	S ^e^_MeOH_ (%)	S ^f^_HA_ (%)	STY ^g^_HA_ (mmol g_cat_^−1^ h^−1^)	Refs.
1	Na-Co/SiO_2_	220	1.9	3	3	12.4	/	26.8	/	73.2	/	[74]
2	Co_3_O_4_	200	2	6	3	28.9	0	53.6	/	19.2	1.6	[75]
3	Co/La_4_Ga_2_O_9_	280	3	3	3	9.6	10.8	52.2	13.7	23.3	/	[76]
4	Na-Co/SiO_2_	250	5	6	3	21.5	26.3	61.2	1.7	10.8	0.47	[77]
5	Mo-Co-K	320	5	3	3	23.5	/	21	70	8.9	/	[78]
6	CoMoC_x_-800 ^h^	180	2	/	3	/	/	/	/	/	0.53	[79]

^a^ Gas hourly space velocity; ^b^ CO_2_ conversion; ^c^ selectivity to carbon monoxide; ^d^ selectivity to hydrocarbon; ^e^ selectivity to methanol; ^f^ selectivity to higher alcohols; ^g^ space–time yield of higher alcohols; and ^h^ tank reactor.

Researchers found that the structure of Co-based catalysts also influenced the carbon chain’s growth ability and methane selectivity. Li et al. [75] synthesized mesoporous-structured Co_3_O_4_ (Co_3_O-m) using mesoporous silica (KIT-6) as a template. It is believed that the formation of long-chain hydrocarbons and alcohols is attributed to Co_3_O_4_ with an ordered mesoporous structure. At the same time, the formation of Co^0^ is also reduced to suppress the formation of undesirable CH_4_.

Furthermore, the modification of Co-based catalysts with metals such as La and Ga can enhance the RWGS reaction’s activity and facilitate the reduction of Co-based catalysts. An et al. [76] obtained a Co/La_4_Ga_2_O_9_ catalyst through the reduction of LaCo_0.5_Ga_0.5_O_3_ perovskite. Unlike other La-based catalysts, strong interactions between Co nanoparticles and La_4_Ga_2_O_9_ were generated on this catalyst. RWGS reactions were carried out on La_4_Ga_2_O_9_, and then the formed CO migrated to Co^0^-Co^2+^ on CoNPs. The metallic Co^0^ absorbed CO dissociatively and hydrogenated to CH_x_. Finally, CO insertion into CH_x_ took place, followed by hydrogenation to produce ethanol. Unfortunately, as the reaction time increased, the yield of alcohols decreased due to the increasing Co^0^/Co^2+^ ratio.

In traditional FTS reactions, the formation of Co_2_C is often considered an important cause of the deactivation of Co-based catalysts. However, recent studies have shown that Co_2_C can promote non-dissociative adsorption and the insertion of CO, improving the selectivity of the oxygen-containing compounds in the product. Sun et al. [77] adjusted the interaction force between Na and Co_2_C by controlling the content of Na. The enhanced interaction significantly improved the dispersion of Co_2_C and reduced the particle size, thereby enhancing the RWGS formation rate and ethanol STY. The adsorption of CO_2_ and CO on Na-Co_2_C active sites also increased, thereby promoting CO insertion to form ethanol. A moderate interaction was obtained at 2 wt% Na, and the ethanol STY reached values as high as 1.1 mmol g^−1^ h^−1^, which was 10 times higher than that without Na.

Researchers found that the synergistic effect between Mo and Co could enhance the C-C coupling ability of Co-based catalysts. Wang et al. [78] prepared Mo-Co-K sulfidation catalysts using activated carbon as a support. The performance of the catalysts was further optimized by adjusting the Co/Mo and K/Mo molar ratios to improve the selectivity of C_2+_OH. The surface Mo^4+^ species was beneficial for CO insertion and carbon chain growth, thereby increasing the selectivity of C_2+_OH. Finally, high CO_2_ conversion (23.7%) and C_2+_OH selectivity (10.0%) were achieved under the conditions of 320 °C, 5.0 MPa, and 3000 mL g_cat_^−1^h^−1^. Wu et al. [79] prepared CoMoC_x_ catalysts using ionic liquid as a precursor. The electronic environment of the catalyst could be adjusted by changing the carbonization temperature. The synergistic effect of Co, Mo_2_C, and Co_6_Mo_6_C_2_ in CoMoC_x_-800 promoted the activation of H_2_ and CO_2_, which was beneficial to C-C coupling.

From the previous works discussed above, the Co-based catalysts generally exhibited lower CO_2_ conversion and high alcohol selectivity compared to other catalysts. The synergy between these Co^0^, CoO, Co^δ+^, and Co_2_C sites is crucial for the formation of alcohols. Thus, the precise regulation of Co hetero sites should be further emphasized for CO_2_ hydrogenation to higher alcohols, by changing the pretreatment conditions, introducing alkali or transition metals, or controlling the interaction between Co and the supports.

### 2.3. Cu-Based Catalysts

Cu is commonly utilized in methanol synthesis and RWGS [70,80,81,82], and modified Cu catalysts are used for the synthesis of higher alcohols from syngas [83,84,85,86,87]. It is widely acknowledged that Cu is responsible for the dissociation of CO to form CO* intermediates, resulting in methanol or CO as the main product. Alkali-modified or FTS (Fe and Co) element-modified Cu-based catalysts yield more high alcohol products by increasing the carbon chain’s growth ability [88]. An intimately cooperated structure with copper enclosed in zeolite can also facilitate the formation of ethanol [89,90]. Some representative catalysts and their performance in CO_2_ hydrogenation to higher alcohols are displayed in Table 4.

**Table 4:** Catalytic performance of HAS from CO_2_ hydrogenation over Cu-based catalysts.

Enter	Catalysts	T (°C)	P (MPa)	GHSV ^a^ /L g^−1^ h^−1^	H_2_/ CO_2_	X ^b^_CO2_ (%)	S ^c^_CO_ (%)	S ^d^_HC_ (%)	S ^e^_MeOH_ (%)	S ^f^_HA_ (%)	STY ^g^_HA_ (mmol g_cat_^−1^h^−1^)	Refs.
1	Cu@ Na-Beta	300	1.3	12	1	7.9	30.5	/	/	69.5	17.1	[89]
2	Cu@m-Beta ^h^	200	4	/	3	17	10	/	20	68	50.13 ^i^	[90]
3	Cu-CoGa_0.4_	220	3	6	3	17.8	2.3	43.5	27.5	23.8	1.35	[91]
4	Na-CuCo-9	330	4	5	1	20.1	26.5	53.8	0.5	26.3	1.09 ^j^	[92]
5	Cs-C_0.8_F_1.0_Z_1.0_	330	5	4.5	3	36.6	/	/	/	19.8	1.47	[93]
6	Cr-CuFe	320	4	6	3	38.4	14.8	/	/	29.2	104.1 ^k^	[94]
7	sp-CuNaFe	310	3	28.8	/	32.3	/	55	/	10	153 ^k^	[95]

^a^ Gas hourly space velocity; ^b^ CO_2_ conversion; ^c^ selectivity to carbon monoxide; ^d^ selectivity to hydrocarbon; ^e^ selectivity to methanol; ^f^ selectivity to higher alcohols; ^g^ space–time yield of higher alcohols; ^h^ tank reactor; ^i^ mmol g_Cu_^−1^h^−1^; ^j^ C_3+_ alcohol space–time yield; and ^k^ mg g_cat_^−1^h^−1^.

Researchers have found that the surrounding environment of copper nanoparticles is also crucial for HAS. Ding et al. [89] embedded 2–5 nm Cu nanoparticles in Na-Beta zeolite with a mesopore structure, and the space–time yield of ethanol reached 398 mg g_cat_^−1^ h^−1^ at 300 °C, 12,000 mL g_cat_^−1^ h^−1^, and 2.1 MPa. Both the surroundings and the reactive centers are important for ethanol production by the rapid bonding of CO_2_* with surface methyl species. Dimitra et al. [90] also used MFI and BEA zeolite to encapsulate Cu nanoparticles to produce mesoporous zeolite catalysts. The introduction of mesopores into zeolite improves conversion and selectivity. Mesoporous Cu-containing Beta zeolite catalysts are more favorable for ethanol production, while non-mesoporous catalysts produce more CO.

**Figure 1:**
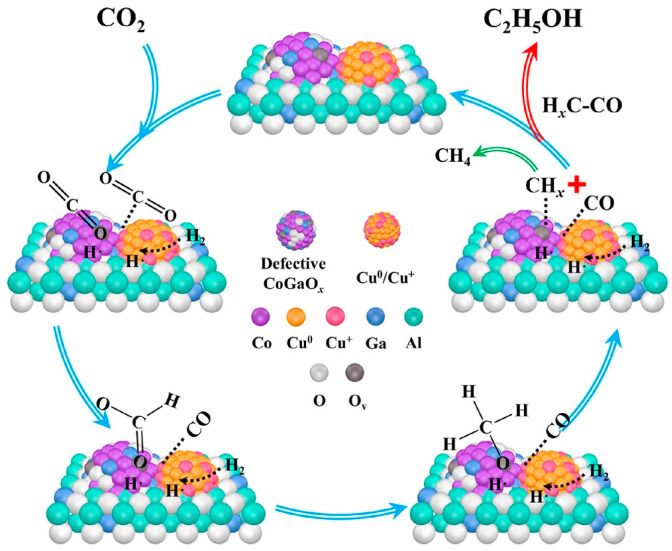
Proposed mechanism for CO_2_ hydrogenation to produce ethanol over Ga-promoted CuCo-based catalysts. Reprinted with permission from Li et al. [91]. Copyright (2022) ACS.

Many studies have been conducted to shift the main product from C_1_OH to C_2+_OH by improving the C-C coupling ability over modified Cu-based catalysts. Li et al. [91] prepared a series of Ga-modified CuCo catalysts with a hydrotalcite structure. Ga species can promote the reduction of Co^3+^ through electronic interactions with Co species in the spinel phase, thereby generating more defective CoGaO_x_ species. At the same time, the strong interaction between Cu and Co species can also be regulated, forming rich Cu-CoGaO_x_ or Cu^+^-CoGaO_x_ interfaces. Figure 1 illustrates the proposed mechanism of CO_2_ hydrogenation to form ethanol. At the beginning of the reaction, CO_2_ molecules are strongly adsorbed on oxygen vacancies of defective CoGaO_x_ species. Afterwards, with the help of dissociated hydrogen species, the carbonate species can further hydrogenate to form formate and methoxy species. Subsequently, the strongly adsorbed methoxy species tend to transform into CH_x_ species on the surface of CoGaO_x_. A large number of Cu^+^ sites at the interface can adsorb gaseous CO from RWGS, which facilitates the insertion of CO on Cu^+^ sites into the CH_x_ generated by adjacent CoGaO_x_ species to form C_2_H_5_O* species, ultimately leading to hydrogenation to produce ethanol. Under the reaction conditions of 220 °C and 3 MPa, the selectivity of ethanol was 23.8%, and the STY of EtOH was 1.35 mmol g_cat_^−1^ h^−1^.

A Cs/Cu/ZnO catalyst was prepared by Wang et al. [96] through the co-deposition of Cs and Cu on a ZnO support. The introduction of Cs deposition created multifunctional sites with a unique structure at the Cu-Cs-ZnO interface, which facilitated the interaction with CO_2_ and predominantly promoted methanol synthesis via the formate pathway. Importantly, this regulation of CHO* binding strength enabled the efficient decomposition of HCOOH into CHO* through the formate pathway while allowing for subsequent hydrogenation into methanol. Furthermore, fine-tuning CHO* binding facilitated the close arrangement of CHO* pairs to promote C-C coupling and ultimately enhance ethanol synthesis.

In order to enhance the C-C coupling ability of Cu-based catalysts, many researchers modified Cu-base catalysts by FTS (Fe and Co) elements with a strong carbon chain growth ability. Jaehoon et al. [92] designed a Na-promoted bimetallic CuCo catalyst (Na-CuCo-y), which could directly hydrogenate CO_2_ to produce C_3+_OH. The metal Co sites were responsible for the dissociation of CO and carbon chain growth, while Cu produced alcohols by inserting CO into the growth chain which occurred in its vicinity. Xu et al. [93] prepared Cs-modified CuFeZn catalysts for efficient higher-alcohol synthesis from direct CO_2_ hydrogenation. A high STY of 73.4 mg g_cat_^−1^ h^−1^ and a C_2+_OH/ROH ratio of 93.8% were achieved due to the interface synergy of Cu-ZnO and Cu-Fe carbides and the regulation of the hydrogenation ability of the catalyst by Cs. In this multi-component catalyst, ferric carbide was responsible for the dissociation of CO (forming alkyl intermediates), Cu was responsible for the non-dissociative activation of CO, and the synergistic effect of the two components (Cu/Fe = 0.8) resulted in a high selectivity to higher alcohols. Fan et al. [94] prepared a Cr-modified CuFe catalyst by the sol–gel method. Higher alcohols were formed on the CuFe catalyst by the CO intermediate route. Firstly, RWGS occurred on the Cu active site to produce CO, and part of CO dissociated and hydrogenated to CH_x_ intermediates on FeC_x_. Then, the CH_x_ intermediate and CO formed higher alcohols by C-C coupling at the Cu-FeC_x_ interface. The addition of Cr enhanced the interaction between Cu and Fe species, promoting the formation of more Cu-FeC_x_ interfaces. Sun et al. [95] obtained a ternary catalyst by sputtering highly active nano-Cu particles onto a Na-modified Fe_3_O_4_ support through a physical sputtering device. The sputtered Cu on NaFe provided high Cu-Na-Fe coordination, resulting in the presence of metal Cu around the Fe_5_C_2_ active site during CO_2_ hydrogenation. The sputtered Cu facilitated the reduction and carbonization of Fe during the reaction, leading to the formation of more Fe_5_C_2_ sites. The unique structure allowed for matching between C-O activation, C-C coupling, and C-O insertion. High-value olefins and ethanol could be produced under mild conditions with a high STY.

In summary, Cu-based catalysts are preferred for CH_3_OH production, and the C-C coupling ability can be enhanced by FTS (Fe and Co) elements. It is important to balance the adsorption of CO/CH_x_ intermediates for C-C coupling. Methanation should also be suppressed to maximize alcohol production. The interaction between Cu and FTS elements should be studied in following works.

### 2.4. Fe-Based Catalysts

Iron has become a well-known candidate catalyst for CO_2_ conversion due to its high activity in RWGS and FTS, where hydrocarbons are the dominant products [97,98,99,100,101]. To facilitate the formation of alcohols, the ability for non-dissociated CO activation should be incorporated into Fe-based catalysts. With this in mind, researchers have studied the hydrogenation of CO_2_ to higher alcohols with Fe-based catalysts. The catalytic properties of representative catalysts are shown in Table 5. Alkali metals (Na, K, Cs, etc.) can not only enhance the adsorption and activation of CO_2_ molecules but also serve as electronic promoters to regulate the types and proportions of the intermediates, making them essential promoters for Fe-based catalysts [102,103,104,105]. Generally, electron-rich Fe sites tend to catalyze the dissociation of adsorbed CO and generate alkyl species, causing a high selectivity to hydrocarbons. After S modification, CO is more likely to be converted into CO* species on the electron-deficient Fe sites [106].

Sun et al. [107] added Na and S promoters to Fe-based catalysts, significantly improving the performance of CO_2_ hydrogenation to higher alcohols. On one hand, the majority of Fe sites tended to convert the strongly adsorbed CO into CH_x_* species under the promotion of Na. On the other hand, CO was more likely to be converted into CO* species on the Fe sites close to S. Different promoters could place Fe sites in different electronic environments, thereby achieving both CO dissociation activation and non-dissociation activation on one metal phase. The FeNaS-0.6 catalyst achieved a CO_2_ conversion rate of 32.0% and a C_2+_OH selectivity of 12.6% under the conditions of 320 °C, 3.0 MPa, and 8000 mL g_cat_^−1^ h^−1^. Zhang et al. [108] prepared Na-modified microsphere Fe_3_O_4_ catalysts using the solvothermal method. The Na promoter is considered to play a crucial role in the formation of iron carbides and the regulation of iron oxide composition (Fe^2+^/Fe^3+^ ratio). At a low pressure (0.5 MPa), the products of CO_2_ hydrogenation are mainly olefins. However, at a high pressure (3.0 MPa), the synergistic effect of iron oxide and iron carbide leads to efficient ethanol generation. Zhang et al. speculated that CHO* is the key intermediate instead of CO on the Na/Fe_3_O_4_ catalyst at a higher reaction pressure. Guo et al. [109] prepared a Mn- and K- modified iron carbide catalyst. The addition of a K promoter can increase the surface C/H ratio of iron carbide catalysts, inhibit deep hydrogenation reactions, and improve ethanol selectivity. Mn acts as a promoter to accelerate CO non-dissociation activation and promote the formation of oxygen-containing intermediates, resulting in higher CO_2_ conversion rates (38.3 to 40.5%) and selectivity for higher alcohols (0 to 10.5%).

**Table 5:** Catalytic performance of HAS from CO_2_ hydrogenation over Fe-based catalysts.

Enter	Catalysts	T (°C)	P (MPa)	GHSV ^a^ /L g^−1^ h^−1^	H_2_ /CO_2_	X ^b^_CO2_ (%)	S ^c^_CO_ (%)	S ^d^_HC_ (%)	S ^e^_MeOH_ (%)	S ^f^_HA_ (%)	STY ^g^_HA_ (mg g_cat_^−1^ h^−1^)	Refs.
1	FeNaS-0.6	320	3	8	/	32.0	20.7	/	/	12.8	78.5	[107]
2	Na/Fe_3_O_4_	300	3.0	2.5	3	30.6	4.1	53.9	5.3	36.7	/	[108]
3	10Mn1K-FeC	300	3.0	6	3	40.5	33.4	55.9	0.2	10.5	/	[109]
4	KFeIn/ Ce-ZrO_2_	300	10.0	4.5	3	29.6	13.4	53.2	4.7	28.7	/	[110]
5	In_2_Fe/ K-Al_2_O_3_	300	2	4	3	36.7	7.4	37.9	2.3	42	/	[111]
6	PdFe-6.9	320	5.0	6	4	35.6	20.7	/	/	18.2	87.8	[112]
7	Na-ZnFe@C	320	5	0.9	3	38.4	7.6	43.2	1.8	20.3	158.1	[113]

^a^ Gas hourly space velocity; ^b^ CO_2_ conversion; ^c^ selectivity to carbon monoxide; ^d^ selectivity to hydrocarbon; ^e^ selectivity to methanol; ^f^ selectivity to higher alcohols; and ^g^ space–time yield of higher alcohols.

**Figure 2:**
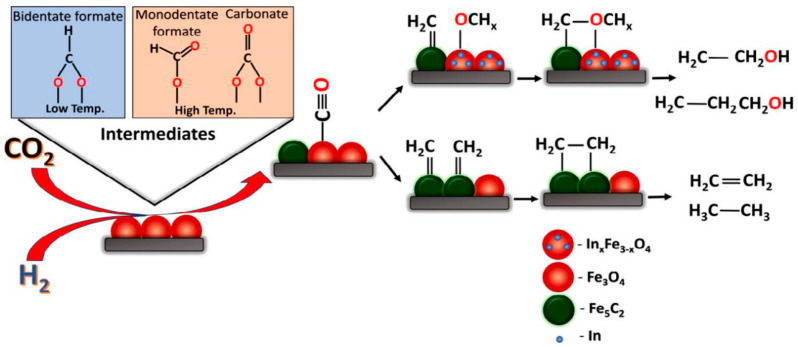
Brief schematic of the mechanism of formation of higher alcohols and other hydrocarbons based on the intermediates observed during an in situ DRIFTS measurement. Reprinted with permission from Peter et al. [111]. Copyright (2022) ACS.

The combination with other metals (e.g., Pd, Cu, In) is usually needed to enhance the CO insertion step to match the alkyl species generated on Fe sites. Xi et al. [110] prepared a series of Ce−ZrO_2_ mixed oxide-supported FeIn catalysts (X-FeIn/Ce−ZrO_2_) with different Fe/(Fe + In) molar ratios (X = 0, 0.18, 0.48, 0.82, 0.88, and 1). The results show that the presence of reduced In_x_O_y_ with oxygen vacancies is essential for CO_2_ hydrogenation to higher alcohols. Peter et al. [111] introduced In into a conventional iron-based catalyst along with K as a promoter, positing that In modification alters the reaction pathway of the traditional Fe_3_O_4_ catalyst (Figure 2). Initially, CO_2_ is converted into CO in the Fe_3_O_4_ phase via the RWGS reaction, followed by hydrocarbon formation on the Fe_x_C_y_ phase. The introduction of induced strain generation facilitates the formation of OCH_x_* intermediates, which subsequently combine with CH_x_ intermediates to form higher alcohols. The addition of K enhances CO_2_ adsorption and higher alcohol selectivity.

The construction of multifunctional interfaces as active sites to achieve the controlled C-C coupling of alkyl with CO*/CH_x_O* is key to improve the performance of Fe-based catalysts for CO_2_ hydrogenation to alcohols. Wang et al. [112] prepared the Fe_2_O_3_ support using the propylene oxide-assisted hydrothermal method and different metal catalysts (M = Pd, Pt, Ag, and Ru) added by the incipient wetness impregnation method. The PdFe alloy is responsible for the RWGS reaction and CO non-dissociative activation, while Fe_5_C_2_ is responsible for CO dissociative activation and carbon chain growth. Due to the appropriate synergistic effect between Pd and Fe, the catalyst achieves a higher alcohol yield of 86.5 mg g_cat_^−1^ h^−1^ with 26.5% selectivity at 300 °C, 5.0 MPa, and 6000 mL g_cat_^−1^ h^−1^. Wu et al. [113] prepared a carbon-based electron buffer layer on the ternary catalytic component ZnO_x_·Fe_5_C_2_·Fe_3_O_4_ to adjust the electron buffer effect, and the electron transfer pathway (ZnO_x_ → Fe species or carbon layer) ensured an appropriate CO* adsorption strength at the catalytic interface. In the ethanol synthesis process, it promotes C-C coupling between CH_x_* and CO* and finally achieves an ultra-high ethanol yield of 366.6 g_EtOH_ kg_cat_^−1^ h^−1^.

In summary, Fe-based catalysts have good application prospects with a high yield of C_2+_ products. After combination with other transition metals or after regulating the electronic property of iron with alkali metals or S, alkyl–*CO coupling can be significantly improved. The balance between the dissociated CO and the non-dissociated CO is considered an effective strategy to enhance alcohol production for Fe-based catalysts, which should be further investigated.

### 2.5. Tandem Catalysts

The concept of tandem catalysis for the hydrogenation of CO/CO_2_ to value-added fuels or products, such as low-carbon olefin [114,115,116,117], aromatic, gasoline, and alcohol, has drawn the attention of many researchers [38,118,119]. For example, a high aromatic selectivity can be achieved by combining the methanol synthesis catalyst with the aromatization zeolite catalyst [120,121,122,123,124,125,126]. The selective synthesis of the target products could cut down the energy consumption for the following separation process, which is crucial for its practical application in industry [127]. Compared with hydrocarbon products, the synthesis of alcohol shows metrics such as a higher atom economy, as the oxygen atom can be incorporated into the alcohol molecule. Wang et al. [128] combined catalysts (ZnO-ZrO_2_|H-MOR|Pt-Sn/SiC) with different functions (methanol synthesis, methanol carbonylation, acetic acid hydrogenation) to produce a high ethanol selectivity. Thus, the rational design of multifunctional tandem catalysts with a controlled reaction channel is crucial for the selective synthesis of the target products.

Due to the complex reaction network, the production of higher alcohols by CO_2_ hydrogenation using conventional catalysts becomes challenging. It is hard to simultaneously consider both CO_2_ activation and C-C coupling in terms of catalytic conversion. Therefore, the reaction-coupling strategy/tandem catalysis emerges as a practical choice for HAS, where the formation, adsorption/desorption, or migration of intermediates (CH_x_O* or CO*) can be manipulated. This reaction-coupling strategy inspired researchers to combine two or more functional catalysts to regulate the reaction network (as depicted in Table 6).

**Table 6:** Catalytic performance of HAS from CO_2_ hydrogenation over tandem catalysts.

Enter	Catalysts	T (°C)	P (MPa)	GHSV ^a^ /L g^−1^ h^−1^	H_2_ /CO_2_	X ^b^_CO2_ (%)	S ^c^_CO_ (%)	S ^d^_HC_ (%)	S ^e^_MeOH_ (%)	S ^f^_HA_ (%)	STY ^g^_HA_ (mmol g_cat_^−1^ h^−1^)	Refs.
1	PdGaCuZnAlK /CuFeAlK	330	8	20	3	54.5	9.7	64.5	5.2	17.0	/	[129]
2	CZK||CFCK	350	6	5	3	32.4	45.3	42.9	5.3	6.5	1.37	[130]
3	Na-Fe@C /KCuZnAl	320	5	/	2.8	39.2	9.4	54.5	4.6	35	/	[131]
4	CuZnAl/K-CuMgZnFe	320	5	6	3	42.3	13.8	67.6	1.3	17.4	2.24	[132]
5	MnCuK-FeC /CuZnAlZr	300	3	6	3	42.1	22.7	60.6	1.2	15.5	/	[133]
6	4.7KCuFeZn /CuZnAlZr	300	5	3	3	29.4	26.1	66.7	2.1	31.2	84.0 ^h^	[134]
7	K-CuZnAl /Zr-CuFe	320	4	24	3	28.8	/	/	/	19.7	195.1 ^h^	[135]
8	Co_2_C||CuZnAl	250	5	12	3	21.2	/	/	/	18.3	2.20	[136]

^a^ Gas hourly space velocity; ^b^ CO_2_ conversion; ^c^ selectivity to carbon monoxide; ^d^ selectivity to hydrocarbon; ^e^ selectivity to methanol; ^f^ selectivity to higher alcohols; ^g^ space–time yield of higher alcohols; and ^h^ mg g_cat_^−1^h^−1^.

In 1998, Yamaguchi et al. [129] first combined the Cu-based catalyst CuZnAlK with Fe-based FeCuAlK, responsible for methanol synthesis and Fischer–Tropsch synthesis, respectively. Compared to single catalysts, the yield of ethanol, C-C bond growth, CO_2_ to CO reduction, and OH insertion were signifyingly enhanced.

Chen et al. [130] combined the RWGS catalyst (K/Cu–Zn) and the Fischer–Tropsch synthesis catalyst (Cu_25_Fe_22_Co_3_K_3_) in a two-stage bed model for HAS. When the reactant gas was fed in, firstly downstream through the CFCK catalyst and then through the CZK catalyst, the STY of higher alcohols was much higher than in the inverse model. It was deemed that the CO generated from the RWGS catalyst was used as the feedstock for subsequent CO hydrogenation over the Cu25Fe22Co3K3 catalyst, thus promoting the production of higher alcohols and other C_2+_ hydrocarbons and/or oxygenates. It was also observed that the yield of C_2+_ hydrocarbons and oxygenates increased, while the selectivity of CO decreased.

Yang et al. [131] prepared a Na-Fe@C catalyst by pyrolyzing the Fe-based MOFs under an inert atmosphere. The selectivity of alkene reached 54.9% for 3%Na-Fe@C, while the ethanol selectivity was only 14.0%. Because the alkali Na metal can donate electron to the Fe-based catalyst, the hydrogenation of alkene to alkane can be hindered by unfavorable H_2_ adsorption. After combination with the CuZnAl catalyst, the selectivity of ethanol increased significantly from 14.0% to 35.0%, while alkene selectivity decreased from 54% to 33.0%. CO_2_ was continuously converted to a CO* intermediate through the RWGS reaction on the CuZnAl catalyst, participating in the subsequent FTS process and ethanol synthesis on the Na-Fe@C catalyst.

Liu et al. [132] prepared K-CuMgZnFe oxides using the co-precipitation method, which was combined with the commercial CuZnAl catalyst into a new type of multifunctional catalyst. CuZnAl was responsible for the produced CO* species, which migrated to the surface of K-CuMgZnFe for HAS. The five catalyst configuration methods were carefully compared to better understand the reaction network, including dual bed, granule stacking, powder mixing, and mortar mixing. It was demonstrated that proximity between the different components is crucial to the migration of chemisorbed CO*. The highest STY of higher alcohols was observed when the above components were combined through powder mixing.

Guo et al. [133] also coupled a Mn-Cu-K-modified FeC catalyst(MnCuK-Fe_5_C_2_) with a CuZnAlZr catalyst to produce higher alcohols. The powder mixing–filling method can improve the selectivity of higher alcohols from 13.5% (MnCuK-FeC alone) to 15.5%. The in situ DRIFT results show that a tandem catalyst promotes the formation of C_2_H_5_O*. Thus, a shorter distance between the two components facilitates the formation of long-chain oxygenated intermediates and the subsequent production of higher alcohols. Therefore, a shorter distance between the two components facilitates the formation of long-chain oxygenated intermediates and subsequently enhances the production of higher alcohols. It is noticeable that the selectivity towards propanol and butanol with more carbon reaches 40% among the total higher alcohol products, which can be ascribed to the co-modification of ferric carbide with transition metal Mn, Cu, and alkaline metal K. The synergistic effect of the multifunctional active sites resulted in the enhanced formation of C_2_H_5_O* and the final higher alcohols.

To investigate the effect of methanol synthesis catalysts, Liu et al. [134] combined CuZnAlZr, ZnZr, and ZnCrAl with the KCuFeZn (KCFZ) catalyst, considering that CH_x_O*/CO* intermediates can be supplied to regulate the reaction network of HAS to C_2+_OH products. It was found that CuZnAlZr with a high RWGS activity and the resulting CO products are most favorable among the methanol synthesis catalysts, with the STY of higher alcohols increasing to 84.0 mg g_cat_^−1^ h^−1^. The promoting effect of ZnZr and ZnCrAl on higher alcohols’ selectivity is, however, limited. It is interesting to note the linear correlation between higher alcohols’ STY and CH_3_OH+CO yield, confirming the importance of CH_x_O*/CO* intermediates in HAS.

Recently, Fan et al. [135] prepared a series of Zr(x)-CuFe oxide catalysts by the co-precipitation method. The selectivity and STY of higher alcohols were 11.9% and 38.2 mg g_cat_^−1^ h^−1^ on the neat Zr (0.03)-CuFe catalyst at 300 °C, 4.0 MPa, and 6000 mL g_cat_^−1^ h^−1^. After combining Zr (0.03)-CuFe and K-CuZnAl, the C_2+_OH selectivity and STY were considerably elevated to 24.5% and 89.1 mg g_cat_^−1^ h^−1^ in the same conditions. The in situ DRIFTS results indicated that K-CuZnAl enhanced the RWGS reaction and accelerated the formation of CO* intermediates. These CO* species rapidly diffused onto the Zr-CuFe catalyst and promoted the formation of higher alcohols through CO* insertion reactions.

Considering the RWGS and C-C coupling capability of the Co_2_C nanoprisms, Sun et al. [136] combined Co_2_C nanoprisms with CuZnAl. After investigating different tandem modes, the STY of higher alcohols reached 2.2 mmol g_cat_^−1^ h^−1^ for the dual-bed Co_2_C||CuZnAl tandem catalyst, where Co_2_C and CuZnAl were configured on the upstream and downstream layer, respectively. The fraction of C_2+_OH in the total alcohol products was 85.5%. Compared with the single Co_2_C catalyst, the yield of higher alcohols tripled for the tandem catalyst. Olefins were first formed on the upstream Co_2_C layer and then diffused downstream to the CuZnAl layer. The R-CH_x_ intermediates were coupled with the CH_x_O intermediates (produced by CuZnAl) to form higher alcohols.

## 3. The Regulation Mechanism of CO_2_ Hydrogenation to Higher Alcohols

Since the latter part of the 20th century, there has been extensive research elucidating the intricate reaction mechanisms involved in CO_2_ hydrogenation. However, the matter of which reaction intermediates are produced during this reaction, such as CHO*, HCOO*, CH_x_*, and CH_3_CO*, is complicated. Consequently, there remains ongoing debate regarding the precise mechanism and pathway for converting CO_2_ into higher alcohols. The CO-mediating mechanism and formate/methoxy-mediating mechanism are widely accepted for HAS.

### 3.1. CO-Mediating Mechanism

**Figure 3:**
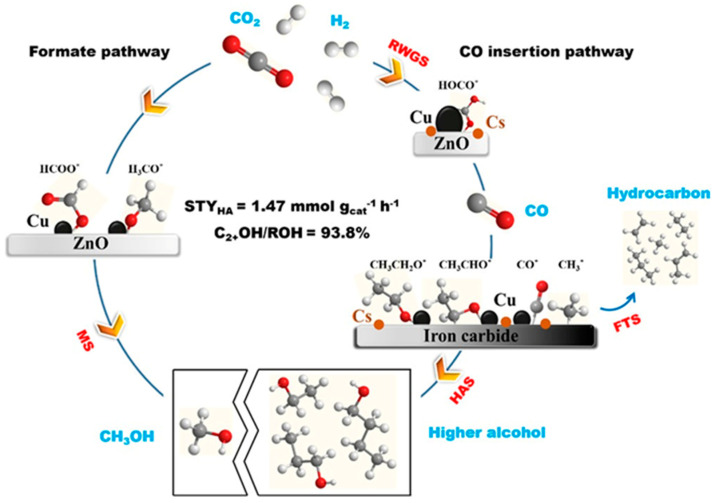
Reaction pathways of CO_2_ hydrogenation over the Cs-CuFeZn catalysts. Reprinted with permission from Xu et al. [93]. Copyright (2020) ACS.

Arakawa et al. [73] proposed the CO-mediating mechanism, whereby the initial CO was formed by the reverse water–gas shift reaction (RWGS). Subsequently, the dissociation of CO produced the CO* species, which was then reacted with H_2_ to produce the *CH_3_ species. Finally, higher alcohols were formed by CO insertion into the alkyl intermediates and hydrogenation. Xu et al. [93] also revealed the CO-mediating mechanism using in situ DRIFTS analysis over the Cs-CuZnFe catalysts. Their findings revealed that, during the course of the reaction, the characteristic peak corresponding to the CO* species was observed first, followed by the adsorption of acetaldehyde and ethoxy intermediates, indicating that the formation of higher alcohols on the catalyst followed a CO insertion mechanism (Figure 3). Methanol steam-reforming experiments demonstrated the difficulty of breaking the C-O bond within methanol, further supporting the preferential formation of alkyl species through CO dissociation.

This reaction mechanism has been well validated in tandem catalytic systems composed of Fe-based and Cu-based catalysts. Among these tandem catalysts, Cu-based catalysts usually play a role in enhancing the RWGS reaction and promoting the generation of oxygen-containing intermediates. While the C-C coupling reaction occurs between oxygen-containing intermediates and alkyl species generated on Fe-based catalysts, further hydrogenation produces higher alcohols. The catalytic performance of HAS is determined by the synergetic effect between the multifunctional components of the tandem catalyst. It is necessary to investigate the product distribution of different sequences and spatial distances between components in order to gain a deeper understanding of the reaction mechanism.

**Figure 4:**
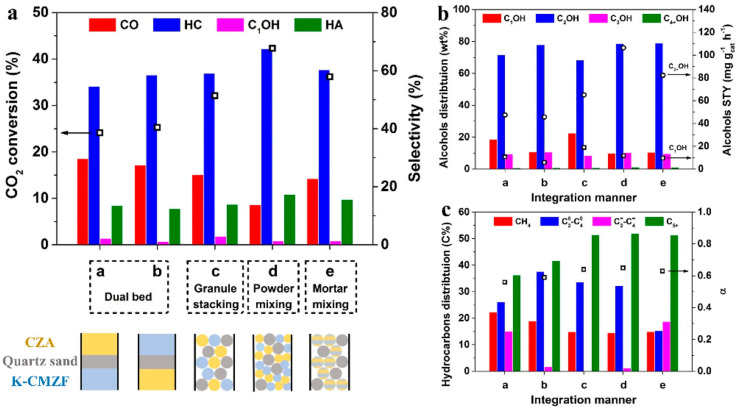
Results of CO_2_ hydrogenation over catalysts packed in different manners. (**a**) CO_2_ conversion and product selectivity, (**b**) alcohol distribution and STY, and (**c**) HC distribution and α over a series of CZA/K-CMZF multifunctional catalysts (CZA/K-CMZF mass ratio of 1) with different integration methods. Reaction conditions: 5 MPa, CO_2_/H_2_ = 1/3, 6 L g_cat_^−1^ h^−1^, and 320 °C. Reprinted with permission from Liu et al. [132]. Copyright (2021) ACS.

Liu et al. [132] fixed the mass ratio of CZA and K-CMZF catalysts at 1:1 and investigated the effects of the five integration methods on the catalysts, as shown in Figure 4. When the catalysts were integrated in a dual-bed manner, the CO_2_ conversion was low. The higher alcohols’ selectivity and STY were also limited (Figure 4b). When shortening the distance of the two components by granule mixing, both CO_2_ conversion and higher alcohols’ STY increased. A closer proximity between the two catalytic components could facilitate the conversion of CO_2_ into higher alcohols through a multi-step reaction channel. For the catalyst with powder mixing, the highest CO_2_ conversion (42.3%), higher alcohols’ selectivity (17.4%), and STY (106.5 mg g_cat_^−1^ h^−1^) with a higher alcohol fraction of 90.2% were obtained. They inferred that adsorbed CO* species rather than gaseous CO were key intermediates for the formation of higher alcohols in this tandem catalysis system. The short distance between the tandem catalysts facilitated the migration of the CO* intermediate.

However, further shortening the distance with mortar mixing leads to a decline in catalytic activity. This is because the CZA component may be poisoned by K from K-CMZF, leading to a decrease in its hydrogenation ability. Consequently, a proper proximity with a certain distance between active sites is most favorable for CO_2_ hydrogenation to higher alcohols.

**Figure 5:**
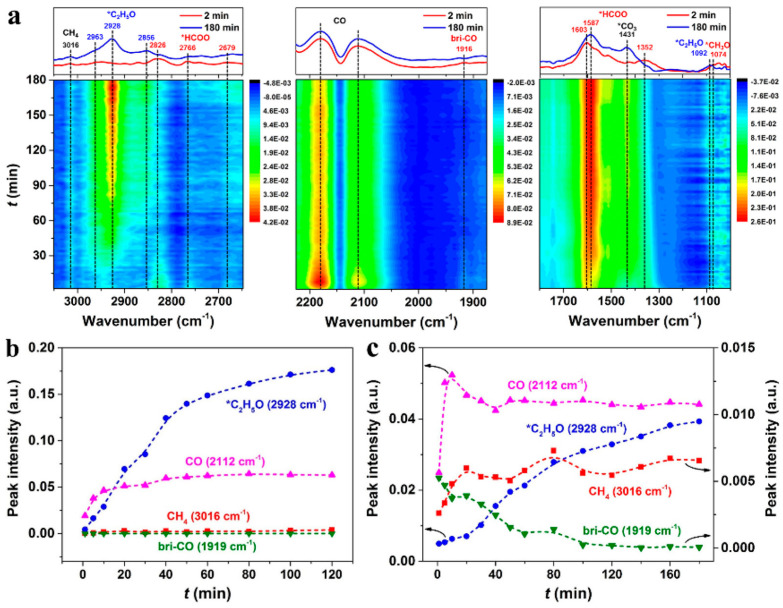
In situ DRIFTS characterization of CO_2_ hydrogenation taken under 0.3 MPa CO_2_/H_2_ flow of 30 mL min^−1^ at 250 °C. (**a**) DRIFTS spectra over CZA/K-CMZF multifunctional catalyst. (**b**,**c**) Dynamic IR peak intensity of CH_4_ (3016 cm^−1^), *C_2_H_5_O (2928 cm^−1^), CO (2112 cm^−1^), and bri-CO (1919 cm^−1^) over (**b**) K-CMZF and (**c**) CZA/K-CMZF catalysts at 250 °C. Reprinted with permission from Liu et al. [132]. Copyright (2021) ACS.

The reaction mechanism of CO_2_ hydrogenation was investigated over a tandem catalyst. As for the sole K-CMZF catalyst, there emerged HCOO*, C_2_H_5_O*, CH_3_CHO*, and CO species as the CO_2_ hydrogenation reaction progressed. The formation of higher alcohols involves a tandem process of the RWGS reaction, CO dissociated to the CH_x_ species, CO insertion, and the hydrogenation of CH_3_CHO*. The successful observation of these intermediates proves the CO-mediated reaction pathway. Similar intermediates are also observed for the CZA/K-CMZF catalyst, indicating the same reaction pathway of CO_2_ hydrogenation to higher alcohols via the CO-mediated reaction pathway. The band of gaseous CO (2112 cm^−1^) on CZA/KCMZF rapidly increases and reaches a maximum at 10 min (Figure 5c), while that on K-CMZF gradually increases and reaches a platform after 60 min (Figure 5b). This suggests that the RWGS is significantly accelerated with the addition of the CZA component.

**Figure 6:**
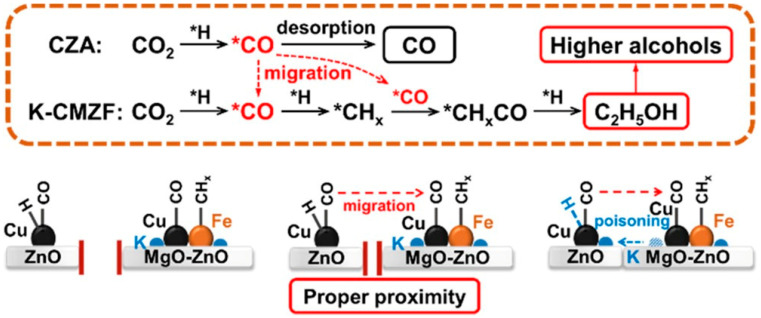
Proposed reaction pathway and proximity effect for CO_2_ hydrogenation to higher alcohols over the CZA/K-CMZF multifunctional catalyst. Reprinted with permission from Liu et al. [132]. Copyright (2021) ACS.

Based on the above research, a reaction pathway was proposed, shown in Figure 6. The CZA component was in favor of CO species (CO*) formation, while the K-CMZF component was in charge of the formation of CH_x_* by the non-dissociation adsorption of CO*. The transformation of CO_x_ to higher alcohols was via the CO-mediating mechanism. The synergy between the two components with a proper mass ratio guaranteed the continuous and rapid transformations of CO_2_ to CO* and CO* to higher alcohols. The rate matching of the above two reactions is important for HAS. CO/CO* is the key intermediate in HAS, and it is of great importance for it to achieve a level of high activity. However, this reaction is considered to be restricted by kinetics rather than thermodynamics due to the low CO concentration in the gas phase, and the coverage of chemisorbed CO (CO*) on the catalyst is also limited. Thus, the proximity between the two components determines the migration of chemisorbed CO* and the sequential synthesis of higher alcohols.

**Figure 7:**
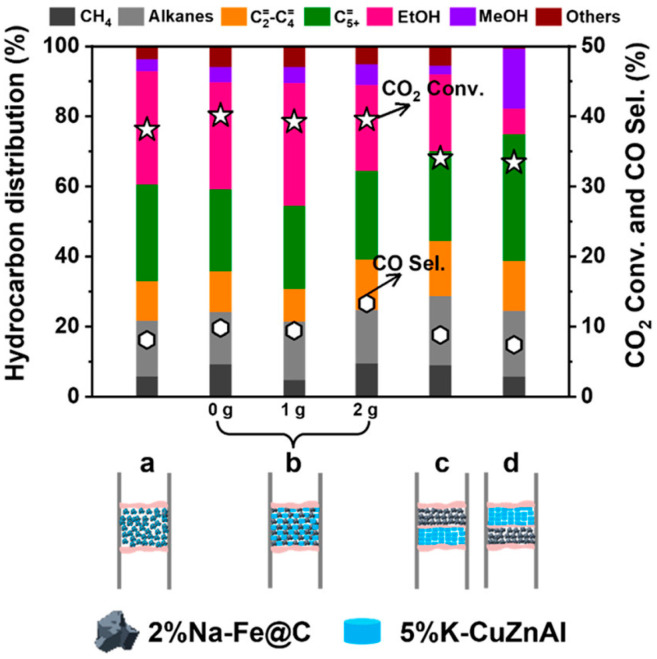
Effect of intimacy modes on catalytic performance. (a) Physical mixing of the catalyst powder, 2% Na-Fe@C/5%-K-CuZnAl. (b) Granule mixing, 2% Na-Fe@C/5% K-CuZnAl with different amounts of quartz sand (0, 1, and 2 g). (c) Dual bed with 2% Na-Fe@C loaded above 5% K-CuZnAl and 2% Na-Fe@C||5% K-CuZnAl. (d) Dual bed with 2% Na-Fe@C loaded below 5% K-CuZnAl and 5%K-CuZnAl||2% Na-Fe@C. Reaction conditions: 320 °C, 5 MPa (25.6% CO_2_, 71.36% H_2_, and 3.04% Ar), 15 mL min^−1^, and time on stream (TOS) = 8 h. Catalyst weight: 0.1 g of Na-Fe@C, 0.1 g of 5%K-CuZnAl, and 1 g of quartz sand. Reprinted with permission from Yang et al. [131]. Copyright (2021) ACS.

Yang et al. [131] also found that the granule-mixed Na-Fe@C/K-CuZnAl catalyst with a short distance achieved higher ethanol selectivity and CO_2_ conversion than other combination methods (Figure 7). A longer distance between the different components of the catalyst led to a decrease in ethanol selectivity, which could be attributed to difficult C-C coupling (CO insertion) to synthesize higher alcohols. It is worth noting that, when the CZA catalyst is placed before NaFe (K-CuZnAl||Na-Fe@C), the selectivity of CH_3_OH is significantly higher than that of other combination methods. Although this configuration mode of the multifunctional catalyst could elevate the partial pressure of CO in the HAS system, the increased distance of the different catalytic components hinders C-C coupling by CO insertion. Moreover, the methanol produced from the upper CuZnAl catalyst travels downstream to cover the surface of the NaFe@C catalyst, which is unfavorable to ethanol production.

**Figure 8:**
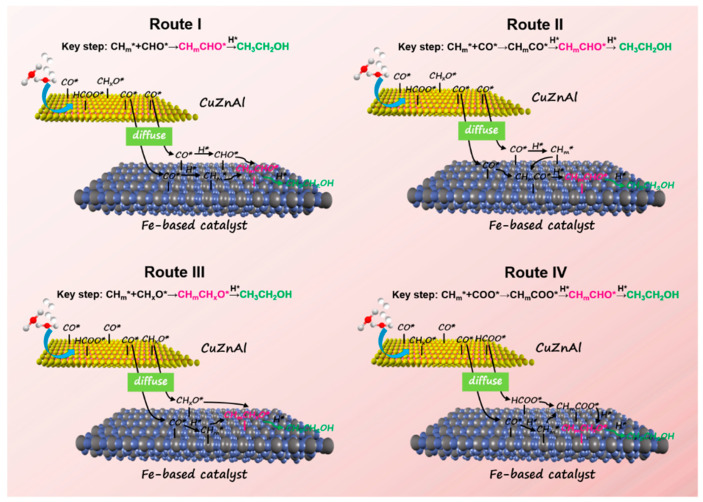
Reaction network for ethanol synthesis from CO_2_ hydrogenation via the Na-Fe@C/K-CuZnAl multifunctional catalyst. Reprinted with permission from Yang et al. [131]. Copyright (2021) ACS.

Based on the results of catalytic performance and in situ characterization, four plausible reaction pathways were proposed for the synthesis of ethanol by tandem catalysis, as shown in Figure 8. In routes I and II, CO* species from the CuZnAl catalyst diffuse onto the surface of the Fe-based catalyst (Fe_5_C_2_ as a mainly active site), where there is dissociation and hydrogenation of the CO* species for CH_m_* formation. Finally, ethanol is formed by different C-C coupling processes. Furthermore, CuZnAl is also a highly efficient catalyst for methanol synthesis, with CH_x_O* or formate as the reaction intermediates. Thus, the formate or CH_x_O* intermediates desorbed from the CuZnAl catalyst can diffuse onto the interface of the Fe-based catalyst for C-C coupling. In route III, the CH_x_O* species from the CuZnAl catalyst couple with the CH_m_* species from the Fe-based catalyst to produce aldehyde species, and ethanol is formed by the subsequent hydrogenation. Different from route III, the species coupling with the CH_m_* species from the CuZnAl catalyst are formate species in route IV. The resulting CH_m_COO* species are hydrogenated to ethanol, with aldehyde as the intermediate (2H* + CH_m_COO*→CH_m_CHO* + OH*).

### 3.2. Formate/Methoxy-Mediated Mechanism

**Figure 9:**
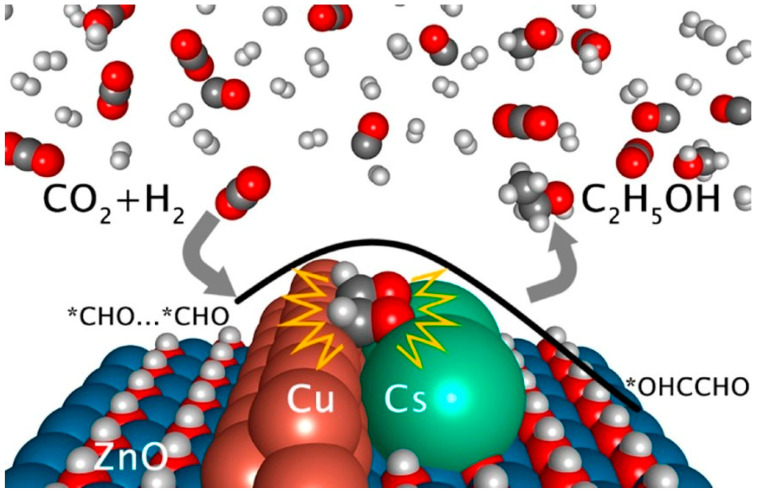
Reaction pathways of CO_2_ hydrogenation over the Cs/Cu/ZnO catalyst. Reprinted with permission from Wang et al. [137]. Copyright (2021) ACS.

He et al. [96] proposed a mechanism involving formate/methoxy intermediates in their study of the Pt/Co_3_O_4_ catalyst. They employed an isotope-labeling technology by adding ^13^CH_3_OH prior to the reaction and observed a peak corresponding to ^13^CH_3_CH_2_OH or CH_3_^13^CH_2_OH with *m*/*z* = 47, indicating that the carbon atom in ethanol may originate from methanol. The authors speculated that water could protonate methanol, leading to its decomposition into the CH_3_* species, which subsequently reacted with CO to form CH_3_CO*. In the following step, the above CH_3_CO* intermediate was hydrogenated to form the target ethanol product. A similar formate/methoxy-mediating mechanism was also put forward by Wang et al. [137]. The adsorption activation of CO_2_ could be facilitated over the Cs-modified catalyst. The energy barrier of HCOO* decomposed into CHO* through the formate pathway was lower than that of CO* hydrogenated into CHO* (Figure 9), thus leading to a higher ethanol yield.

**Figure 10:**
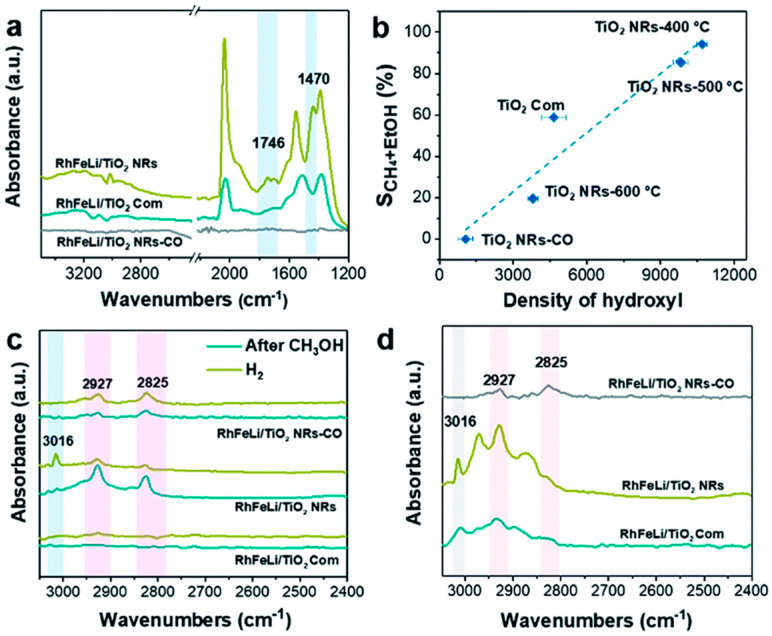
(**a**) In situ DRIFTS of 2.5 wt% RhFeLi/TiO_2_ NRs, 2.5 wt% RhFeLi/TiO_2_ Com, and 2.5 wt% RhFeLi/TiO_2_ NRs-CO under a CO_2_ + H_2_ + Ar (CO_2_:H_2_ = 1:3) atmosphere at 250 °C. (**b**) The total selectivity to CH_4_ and ethanol as a function of the peak area of hydroxyls normalized by the SBET of the samples obtained from 2.5 wt% RhFeLi supported on different TiO_2_ supports. (**c**) In situ DRIFTS of 2.5 wt% RhFeLi/TiO_2_ NRs, 2.5 wt% RhFeLi/TiO_2_ Com, and 2.5 wt% RhFeLi/TiO_2_ NRs-CO after CH_3_OH + Ar adsorption, followed by H_2_ adsorption at 250 °C. (**d**) In situ DRIFTS of 2.5 wt% RhFeLi/TiO_2_ NRs, 2.5 wt% RhFeLi/TiO_2_ Com, and 2.5 wt% RhFeLi/TiO_2_ NRs-CO under a CO + H_2_ + Ar (CO:H_2_ = 1:2) atmosphere at 250 °C. Reprinted with permission from Yang et al. [60]. Copyright (2019) Royal Society of Chemistry.

Yang et al. [60] proposed the same pathway when studying the RhFeLi/TiO_2_ NR catalyst. They believed that the CH_3_* species originated from formic acid/methoxy species and found a linear relationship between the CH_3_* species and the hydroxyl content on the TiO_2_ support (Figure 10b). They conducted reactions of CH_3_OH and H_2_ over RhFeLi/TiO_2_ and investigated the intermediates by in situ DRIFTS. After the feeding of CH_3_OH, all the catalysts showed peaks at 2825 and 2927 cm^−1^ related to the CH_3_O* species. After the feeding of H_2_, the peak distribution of RhFeLi/TiO_2_ did not show significant changes. In contrast, the peak intensity of CH_3_O* in RhFeLi/TiO_2_ NRs decreased, while a CH_4_ peak appeared at 3016 cm^−1^ (Figure 10c). They inferred that the presence of surface hydroxyl groups on TiO_2_ was beneficial for stabilizing formic acid intermediates and breaking C-O bonds in methoxy species. Therefore, hydroxyl groups can protonate methoxy groups, similar to the protonation of methanol in water.

## 4. Effect of the Reaction Conditions on the Catalytic Performance of CO_2_ Hydrogenation

Due to the complex reaction network of CO_2_ hydrogenation, the reaction conditions have a significant impact on HAS, including the reaction temperature, the reaction pressure, the space velocity, and the H_2_/CO_2_ ratio. The stability of the multifunctional catalyst should also be evaluated before further industrial application.

### 4.1. Reaction Temperature

**Figure 11:**
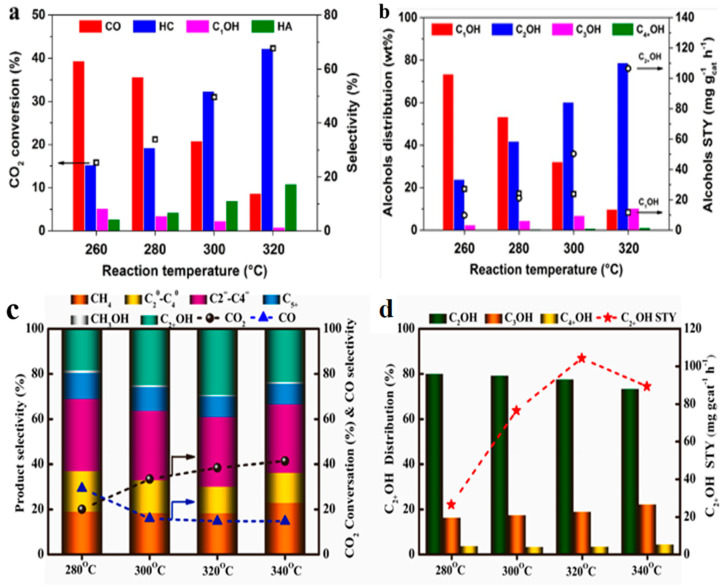
CO_2_ conversion and product selectivity over the CZA/K-CMZF multifunctional catalyst (CZA/K-CMZF mass ratio of 1, powder mixing) at different reaction temperatures (**a**,**b**). Reprinted with permission from Liu et al. [132]. Copyright (2021) ACS. Influence of reaction temperature (**c**,**d**) on the catalytic performance of the Cr (1%)-CuFe catalyst in CO_2_ hydrogenation to higher alcohols. Reprinted with permission from Fan et al. [94]. Copyright (2023) Elsevier.

The catalytic performance of CO_2_ hydrogenation is related to the reaction temperature. Generally, a high temperature can increase the conversion of CO_2_ and the selectivity of C_2+_OH. This is due to CO_2_ activation being promoted by increasing the temperature. However, when the reaction temperature exceeds 320 °C, the CO* intermediate is prone to decompose rapidly to produce more CH_x_* species, which will be further hydrogenated to methane or C_2+_ hydrocarbons. Liu et al. [132] discovered that a high temperature could benefit the formation of higher alcohols and hydrocarbon products, as shown in Figure 11a,b. With the elevation of the temperature from 260 °C to 320 °C, the conversion of carbon dioxide increased significantly from 15.8% to 42.3%. The selectivity of C_2+_OH increased from 4.3% to 17.4%, and the STY of C_2+_OH increased from 9.8 mg g_cat_^−1^ h^−1^ to 106.5 mg g_cat_^−1^ h^−1^. However, CO selectivity decreased from 63.0% to 13.8%. Fan et al. [94] also found that a high temperature could increase the conversion of CO_2_. With the elevation of temperature from 280 °C to 320 °C, the selectivity and STY of C_2+_OH increased from 18.3% and 26.4 mg g_cat_^−1^ h^−1^ to 29.2% and 104.1 mg g_cat_^−1^ h^−1^, respectively (Figure 11c,d). However, a further increase in the reaction temperature to 340 °C led to a decrease in both C_2+_OH selectivity and STY to 23.6% and 89.2 mg g_cat_^−1^ h^−1^, respectively. The generation of more hydrocarbon products, such as methane, light alkanes, and alkenes, was also observed.

### 4.2. Reaction Pressure

**Figure 12:**
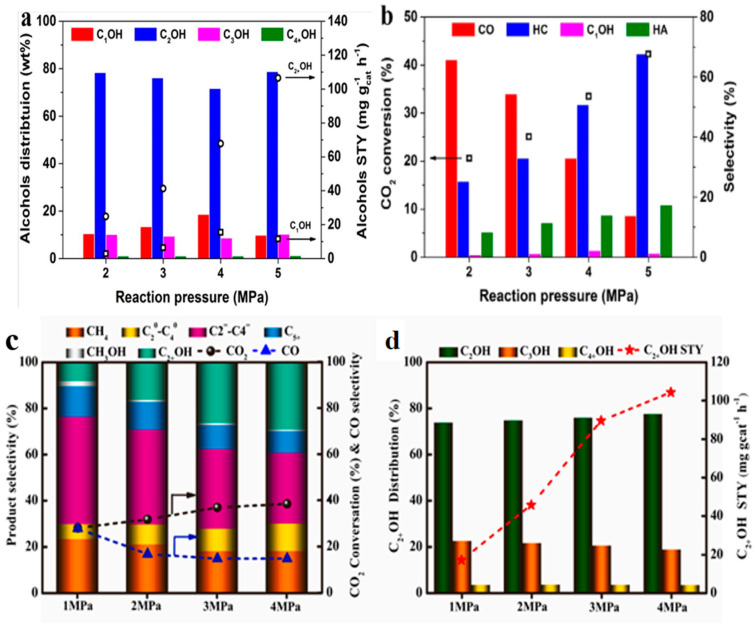
CO_2_ conversion and product selectivity over the CZA/K-CMZF multifunctional catalyst (CZA/K-CMZF mass ratio of 1, powder mixing) at different reaction pressures (**a**,**b**). Reprinted with permission from Liu et al. [132]. Copyright (2021) ACS. Influence of reaction pressure (**c**,**d**) on the catalytic performance of the Cr (1%)-CuFe catalyst in CO_2_ hydrogenation to higher alcohols. Reprinted with permission from Fan et al. [94]. Copyright (2023) Elsevier.

Increasing the reaction pressure can increase CO_2_ conversion and the selectivity and STY of C_2+_OH but decrease CO selectivity because the number of molecules decreases in higher-alcohol synthesis. It is reasonable to conclude that a high pressure facilitates HAS and the formation of both alcohols and hydrocarbons, according to Le Chatelier’s principle. Liu et al. [132] discovered that, when the pressure increased from 2 to 5 MPa, the CO conversion rate improved remarkably, from 33.7% to 86.0%. In addition, CO_2_ conversion was improved, accompanied by the rapid consumption of produced CO. As a result, the multifunctional catalyst exhibits the highest CO_2_ conversion of 42.3% and the lowest CO selectivity of 13.8% at 5 MPa. Meanwhile, the selectivity and STY of high alcohols also increases (Figure 12a,b). Fan et al. [94] found that, with the elevation of the pressure from 1 MPa to 4 MPa, CO_2_ conversion increased from 28.3% to 38.4%. The selectivity and STY of C_2+_OH gradually increased from 7.3% and 17.4 mg g_cat_^−1^ h^−1^ to 29.2% and 104.1 mg g_cat_^−1^ h^−1^, respectively, while CO selectivity decreased from 29.4% to 14.8% (Figure 12c,d).

### 4.3. Space Velocity

In the reaction of carbon dioxide hydrogenation to produce higher alcohols, the variation in space velocity has an important impact on the reaction conversion rate and the selectivity of higher alcohols. As the space velocity increases, the contact time between the catalyst and the feed gas decreases, which is not conducive to the conversion of CO_2_ and the growth of carbon chains. On the contrary, a prolonged residence time promotes the conversion of CO_2_ and the growth of carbon chains and improves the selectivity of higher alcohols. Therefore, a suitable space velocity needs to be selected in the reaction of carbon dioxide hydrogenation to produce higher alcohols.

**Figure 13:**
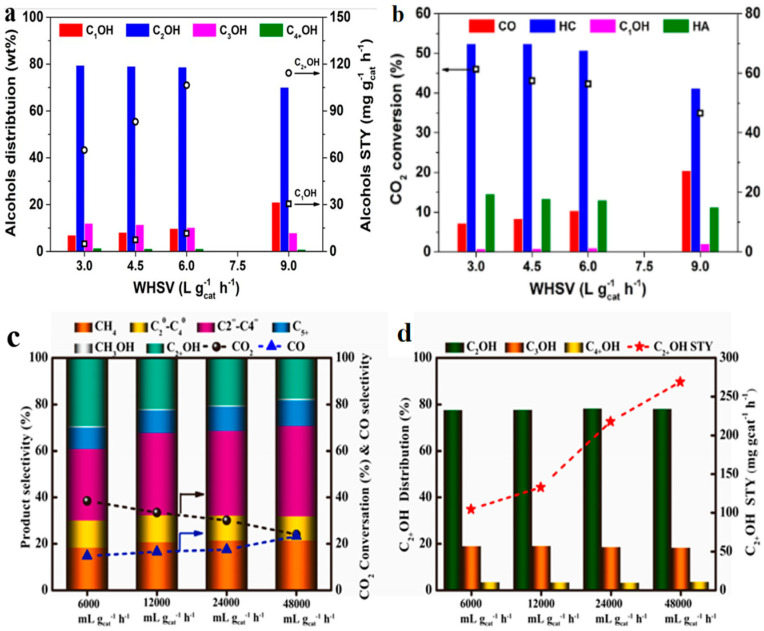
CO_2_ conversion and product selectivity over the CZA/K-CMZF multifunctional catalyst (CZA/K-CMZF mass ratio of 1, powder mixing) at different space velocities (**a**,**b**). Reprinted with permission from Liu et al. [132]. Copyright (2021) ACS. Influence of space velocity (**c**,**d**) on the catalytic performance of the Cr (1%)-CuFe catalyst in CO_2_ hydrogenation to higher alcohols. Reprinted with permission from Fan et al. [94]. Copyright (2023) Elsevier.

Liu et al. [132] discovered that, with the space velocity increasing from 3000 mL g^−1^ h^−1^ to 9000 mL g^−1^ h^−1^, the CO_2_ conversion and selectivity of C_2+_OH gradually decreased from 46.0% and 19.5% to 34.9% and 15.0%, respectively (Figure 13a,b). Fan et al. [94] found that, with the space velocity increasing from 6000 mL g^−1^ h^−1^ to 48,000 mL g^−1^ h^−1^, the CO_2_ conversion and selectivity of C_2+_OH gradually decreased from 38.4% and 29.2% to 24.0% and 17.5%, respectively (Figure 13c,d). The STY of C_2+_OH gradually increased from 104.1 mg g_cat_^−1^ h^−1^ to 268.5 mg g_cat_^−1^ h^−1^.

**Figure 14:**
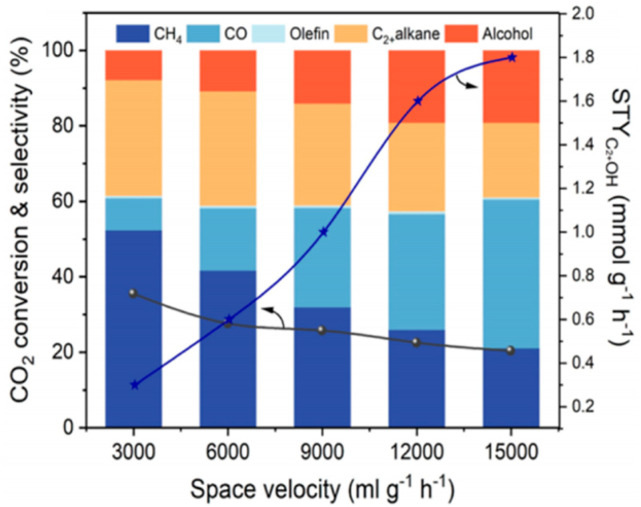
Catalytic performance over Co_2_C||CuZnAl at different space velocities. Reprinted with permission from Sun et al. [136]. Copyright (2023) ACS.

However, Sun et al. [136] observed a contradictory trend: as the space velocity increased from 3000 mL g^−1^ h^−1^ to 15,000 mL g^−1^ h^−1^, the selectivity of hydrocarbon dropped considerably, while the selectivity of CO and higher alcohols gradually increased, which could be ascribed to the avoidance of excessive hydrogenation (Figure 14).

### 4.4. H_2_/CO_2_ Ratio

The H_2_/CO_2_ ratio is an important parameter in CO_2_ hydrogenation to produce higher alcohols. As the H_2_/CO_2_ ratio increases, the conversion of CO_2_ increases. An et al. [76] studied the changes in product selectivity of the Co/La_4_Ga_2_O_9_ catalyst with H_2_/CO_2_ ratios of 3/1, 4/1, and 5/1 after a 12 h reaction. The results showed that, as the H_2_/CO_2_ ratio increased from 3/1 to 5/1, the CO_2_ conversion rate decreased from 7% to 2%, while the ethanol selectivity decreased from 64% to 28%, due to the competition between CO and H_2_ adsorption on the Co NPs surface. Under a lower H_2_/CO_2_ ratio, CO is more likely to adsorb on the Co NPs surface, thus improving ethanol selectivity. However, with a higher H_2_/CO_2_ ratio, H_2_ is more likely to adsorb on the Co NPs surface, reducing CO adsorption and lowering ethanol selectivity; thus, the selectivity of CO is increased. Cui et al. [138] studied the performance of the bimetallic Ru_3_(CO)_12_-Co_4_(CO)_12_ catalyst. The results showed that, when the H_2_/CO_2_ ratio was increased from 1/2 to 2/1, the selectivity of higher alcohols increased from 79.5% to 90.8%; however, when the H_2_/CO_2_ ratio increased further to 3/1, the selectivity of higher alcohols decreased to 67.2%. This indicates that an appropriate H_2_/CO_2_ ratio should be chosen to increase the selectivity of higher alcohols.

### 4.5. Relative Humidity

Water will inevitably be generated during the hydrogenation of CO_2_. The relative humidity may cause the deactivation of catalysts, but the introduction of water in the reaction system could facilitate the formation of higher alcohols. As discussed in Section 3.2, water plays a crucial role in the formate/methoxy-mediated mechanism. The presence of suitable water in a reaction system can enhance selectivity to higher alcohols. He [96] found that water could promote the reaction kinetically. They speculated that water could protonate methanol, which was easily dissociated into *CH_3_, OH*, and H* species, thereby facilitating the reaction. Rodriguez [139] studied the CO_2_ hydrogenation reaction on the Pt/CeO_x_/TiO_2_(110) catalyst under CO_2_/H_2_ and CO_2_/H_2_/H_2_O feeds, respectively. The addition of small quantities of water vapor significantly enhanced the surface coverages of C-containing species (CH_3_O*, HCOO*, *CO_3_, *CH_x_) and alcohol production. The DFT simulations suggested that water increased the CO_2_–support interaction and facilitated the hydrogenation of CO_2_, favoring the formation of the key intermediate CH_3_O at the Pt/CeO_x_ interface.

**Figure 15:**
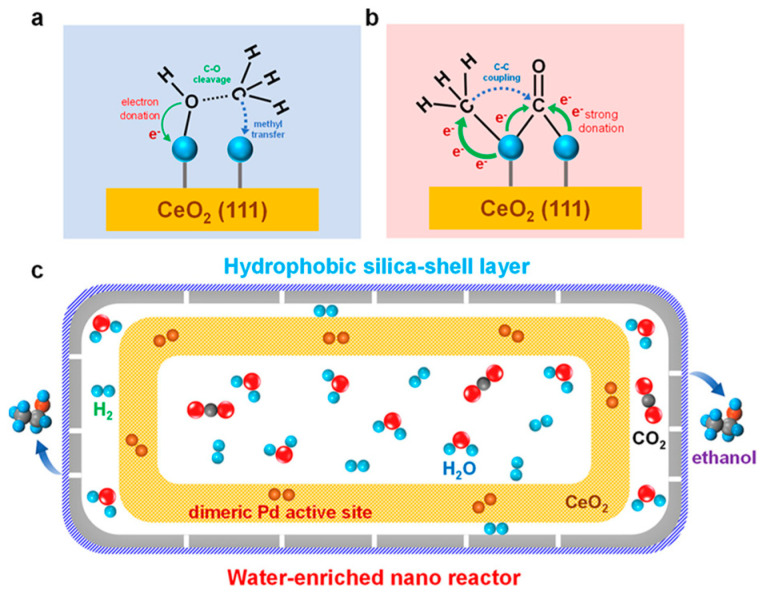
Proposed mechanism for favorable C-O bond cleavage (**a**) and C-C coupling (**b**) over the dimeric Pd active sites. (**c**) Model presenting the in situ generated water enriched in a nano reactor with a hydrophobic silica shell layer. Reprinted with permission from Liu et al. [62]. Copyright (2023) ACS.

In addition, researchers also found that in situ generated water enriched in a nano reactor provided a high yield of ethanol. Liu [62] found that dual Pd sites could be stabilized through the water formed in situ in the nano reactor with a hydrophobic shell layer, and the selectivity to ethanol reached 98.7% (Figure 15). The DFT calculations confirmed that the presence of hydroxyl (OH) with a water-enriched environment remarkably enhanced the energy barrier of Pd atoms’ migration, which thereby stabilized the dimeric Pd active sites for ethanol synthesis.

### 4.6. Catalyst Stability

**Figure 16:**
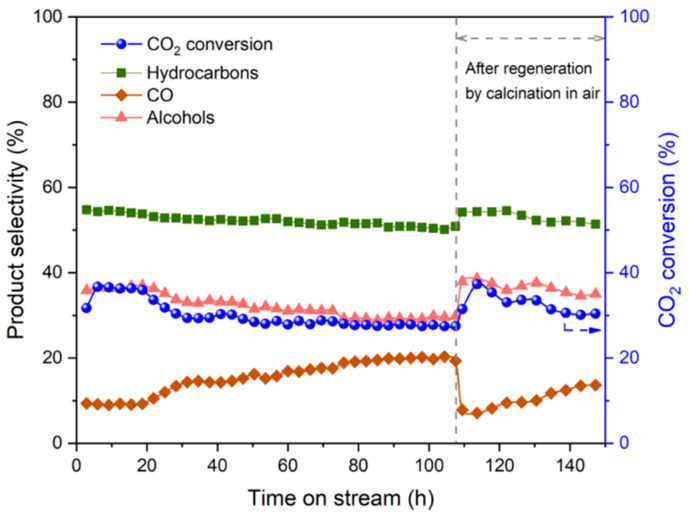
CO_2_ conversion and product selectivity vs. runtime for the K-0.82-FeIn/Ce−ZrO_2__900 (with the presence of SiC_900) catalyst. Reprinted with permission from Xi et al. [110]. Copyright (2021) ACS.

**Figure 17:**
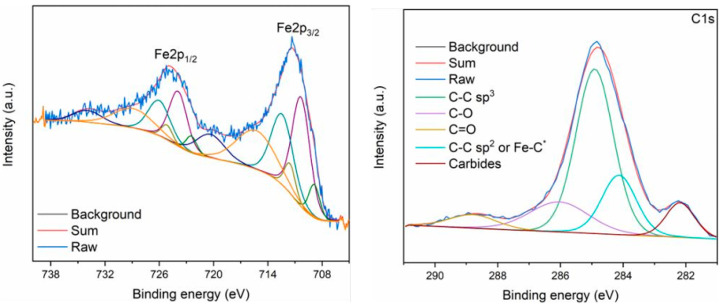
XPS spectra (Fe2p, C1s) of K-0.82-FeIn/Ce-ZrO_2__900 after the reaction. Reprinted with permission from Xi et al. [110]. Copyright (2021) ACS.

In addition to the above reaction conditions, it is also important to conduct long-term tests to investigate the stability of the catalyst, which provides useful insights into the development of catalysts for industrial applications. Xi et al. [110] discovered that, in the initial stage (0–20 h), the CO_2_ conversion (36.3%) was nearly constant and then slowly dropped to 27.8% after 100 h TOS. Product selectivity changed gradually during the runtime, showing an increase in CO selectivity (9.3% at 20 h to 20.2% at 100 h) and a slight reduction in alcohol selectivity (36.3% at 20 h to 29.8% at 100 h). Specifically, the selectivity of higher alcohols decreased slightly from 30.6% at 20 h to 25.4% at 100 h, while the higher alcohols’ content in alcohol fractions remained relatively stable in the range of 84.4–86.5% during the reaction. After the stability experiment (110 h), the spent catalyst was taken out of the reactor for calcination in air (500 °C, 3 h) and then reloaded to the reactor for a regeneration test. To compare its performance with that of a fresh catalyst, CO pretreatment was conducted. The initial activity of the regenerated catalyst was close to that of a fresh catalyst (Figure 16). These results indicate that the deactivation of catalysts is due to the deposition of carbonaceous compounds (e.g., graphitic carbon, amorphous carbon, and coke) during the activity test. The XPS spectra of K-0.82-FeIn/Ce-ZrO_2__900 showed that the peak related to coke formation (C-C sp^2^ or Fe-C*) was much higher (17.86% vs. 3.79%) for the spent catalysts, suggesting that coke deposited on the catalyst surface during the reaction. The DTG profile of the spent catalyst showed considerable weight loss at around 400 °C, which also verified the formation of coke. Furthermore, they also considered the catalyst’s evolution during the reaction in terms of deactivation and whether the formation of In^0^ and iron oxides could be the reason for catalyst deactivation, but these possibilities were ruled out experimentally (Figure 17).

**Figure 18:**
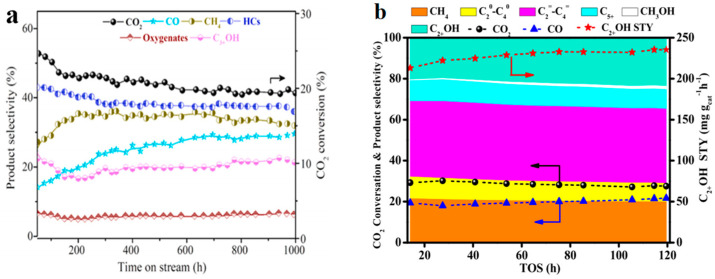
(**a**) Long-term stability test of the Na–CuCo-9 catalyst during CO_2_ hydrogenation. Reprinted with permission from Kim et al. [92]. Copyright (2024) Elsevier. (**b**) Catalytic stability test of the Cr (1%)-CuFe catalyst in CO_2_ hydrogenation to higher alcohols. Reprinted with permission from Fan et al. [94]. Copyright (2023) Elsevier.

Kim et al. [92] studied the long-term stability of the Na-CuCo-9 catalyst, as shown in Figure 18a. After a slight decrease in CO_2_ conversion and C_3+_OH selectivity during the initial 125 h of the on-stream reaction, the catalytic conversion and selectivity were well maintained at 20% and 22%, respectively. The high stability of the catalyst was still maintained after the 1000 h on-stream reaction, indicating good resistance toward carbon deposition and active-site sintering by the interconversion between Cu^0^ and Cu_2_^(1+)^ O. Fan et al. [94] found that the Cr (1%)-CuFe catalyst also presented a high catalytic stability in CO_2_ hydrogenation to higher alcohols. After 120 h TOS, the CO_2_ conversion and selectivity and STY of C_2+_OH, were well maintained at around 35.8%, 23.3%, and 82.5 mg g_cat_^−1^ h^−1^, respectively (Figure 18b).

The techno-economic assessment and process design are important for understanding the effect of catalytic performances (i.e., CO_2_ conversion, product selectivity, and product yield) on the process performance. Kim et al. [140] studied the influence of the operating temperature and pressure on the energy consumption and unit production cost for the process of CO_2_ hydrogenation to methanol. It was found that, with the increase in the operating temperature, methanol selectivity generally decreased, whereas CO_2_ conversion and methanol yield generally increased. Moreover, the increase in the operating pressure improved the CO_2_ conversion, methanol selectivity, and methanol yield. The growth of CO_2_ conversion is beneficial to reducing the amounts of unreacted syngas, which can lead to savings in the heater’s energy consumption during the recycling stage. The increase in the methanol yield is favorable to improving methanol production, which can reduce the unit production cost of methanol.

In addition, the conceptual design and technoeconomic evaluation of processes of CO_2_ hydrogenation to higher alcohols are essential. Unfortunately, relevant research on the systematic modeling of the aforementioned process is still limited. Nevertheless, in the work of Dutta et al. [141,142], they proposed the syngas-to-high alcohol process and implemented a detailed economic assessment. As a result, the capital cost of the alcohol separation unit accounts for more than 25% of the total capital cost, which can provide an important reference for the economic analysis of the process of CO_2_ hydrogenation to high alcohol.

In summary, different reaction conditions exert distinct effects on different multi-component catalysts, so it is hard to draw a unanimous conclusion due to the complex reaction network. The matching of reaction conditions with the multifunctional catalysts is crucial to the HAS performance. Further investigation is warranted to ascertain the optimal reaction conditions. Given these points, developing more efficient catalysts or optimizing the operating conditions to improve the selectivity of the targeted products could contribute to reducing the separation cost and the associated total capital cost of the overall process. In addition, the long-term evaluation of the catalyst is also needed to study the deactivation mechanism of the catalyst for practical applications.

## 5. Conclusions and Future Perspectives

The hydrogenation of CO_2_ to higher alcohols is of significant importance for the conversion of CO_2_ into value-added chemicals. This route also holds the potential to address the greenhouse effect caused by excessive CO_2_ emissions. The hydrogenation of CO_2_ to higher alcohols remains a major challenge, due to the complexity of the reaction and the existence of many by-products such as CO, CH_4_, and other C_2+_ hydrocarbons. In this review, we summarized the latest progress in research on CO_2_ hydrogenation to higher alcohols, emphasizing the state-of-the-art working strategy regarding multifunctional catalysis. Noble metal catalysts generally show a high selectivity for C_2+_OH but have a low CO_2_ conversion. The high loading of noble metal catalysts also makes these catalysts expensive. Non-noble metal catalysts generally have a low selectivity for alcohols and make it necessary to introduce more functional active sites. In comparison, Co-based catalysts can inhibit the conversion of CO_2_ to CO; however, due to their strong methanation ability, the selectivity for C_2+_OH is still low. Cu- and Fe-based catalysts have a relatively high CO_2_ conversion and a high alcohol yield. Considering the cost of catalysts, Cu- and Fe-based catalysts show good prospects. The reaction-coupling strategy holds great potential to regulate the reaction network; thus, the tandem catalyst demonstrates considerable promise for the synthesis of higher alcohols. With advancements in the study of catalysts’ design and reaction mechanism, these practical issues are expected to be addressed.

Firstly, considering the thermodynamics and kinetics of the existing reactions in HAS, the matching of different kinds of components for multifunctional catalysis is crucial. Thus, the rational design of the active sites of the multifunctional catalyst should be highlighted. The functional matching between catalysts with different functions can be achieved through a genetic algorithm and artificial intelligence screening. DFT calculations and molecular dynamic simulations can be applied to better understand the reaction mechanism, which is important for the design of catalyst composition and structure in HAS.

Secondly, due to the existence of various possible reaction pathways, the reaction mechanism of multifunctional catalysis is still far from clear. Key active sites and intermediates during HAS should be identified using in situ characterization techniques, such as IR, XPS, XRD, and TEM. The catalytic structure–performance relationship should be further revealed.

Thirdly, to control the transport of intermediates or products and, thus, the reaction sequence, the appropriate spatial arrangement of different components holds potential to avoid by-product formation. The kinetic matching of cascade reaction steps, such as C-C coupling and CO insertion, are key issues guiding the selection of each catalyst component and the construction of an efficient catalysis system. For this purpose, the rational arrangement of different functional components in a mesoscale with controllable proximity deserves more research. The transportation of intermediates/products in sequence can be manipulated.

Lastly, novel reactors, such as nano-reactors or membrane reactors, can contribute to the optimization of the reaction through adjusting the surroundings or kinetics of HAS. The Cu@Na-Beta nano-reactor reached a high STY of 398 mg g_cat_^−1^ h^−1^, as the HAS was promoted by the confined surroundings of reactive centers in zeolitic frameworks [89]. A water-enriched nano-reactor with dual Pd active sites exhibited high ethanol selectivity (98.7%) by breaking the reaction restriction [143]. Membrane reactors can remove by-products (e.g., water) in situ through selective separation to effectively enhance the yield of higher alcohols. Novel reactors hold a great possibility to overcome the thermodynamic equilibrium limitation.

Multifunctional catalysis is expected to play an important role in the direct synthesis of value-added products in C1 chemistry through enabling the development of new reaction channels with precise control. Future advances are expected for HAS. We anticipate that this review will provide an inspiration for the ongoing development of novel forms of catalysis, and we look forward to the emergence of innovative research efforts aimed at addressing the challenges associated with CO_2_ hydrogenation to higher alcohols.

## Figures and Tables

**Table 1:** The main and side reactions and ΔG_298K_, ΔH_298K_, and K_298K_ of the CO_2_ hydrogenation system.

Reaction Equation	ΔG_298K_ (kJ/mol)	ΔH_298K_ (kJ/mol)	K_298K_
CO_2_ + H_2_ ↔ CO + H_2_O	28.6	41.1	9.67 × 10^−6^
CO_2_ + 4H_2_ ↔ CH_4_ + 2H_2_O	−113.5	−165.0	7.79 × 10^19^
n CO_2_ + (3n + 1) H_2_ ↔ C_n_H_2n+2_ + 2n H_2_O	/	/	/
n CO_2_ + 3n H_2_ ↔ C_n_H_2n_ + 2n H_2_O	/	/	/
CO_2_ +3H_2_ ↔ CH_3_OH + H_2_O	3.5	−49.3	2.45 × 10^−1^
2CO_2_ + 6H_2_ ↔ C_2_H_5_OH +3H_2_O	−32.4	−86.7	4.70 × 10^5^
n CO_2_ + 3n H_2_ ↔ C_n_H_2n+1_OH + (2n − 1) H_2_O	/	/	/

## Data Availability

Data will be made available upon request.
